# Mortality Following Simultaneous Versus Staged Bilateral Total Knee Arthroplasty: A Systematic Review and Meta-Analysis

**DOI:** 10.7759/cureus.50823

**Published:** 2023-12-20

**Authors:** Abbas M Alshaikh, Naif M Alshaeri, Rawaa Jamal, Osama F Almaghthawi, Mithaq M Al Eid, Ziyad S Alfageeh, Anas M Alturkistani, Abdalla Mohamed Bakr Ali

**Affiliations:** 1 Orthopaedics, King Abdullah Medical Complex, Jeddah, SAU; 2 Orthopaedics, South Al-Qunfudhah General Hospital, Al Qunfudhah, SAU; 3 Medicine, Umm Al-Qura University, Jeddah, SAU; 4 Medicine, Taibah University, Madinah, SAU; 5 Medicine, University of Sharjah, Sharjah, ARE; 6 Medicine, University of Jeddah, Jeddah, SAU; 7 Medicine, Jeddah University, Jeddah, SAU; 8 General Surgery, Sohag University, Sohag, EGY

**Keywords:** meta-analysis, mortality, staged, simultaneous, bilateral total knee arthroplasty

## Abstract

Bilateral total knee arthroplasty (BTKA) is a common intervention for bilateral knee osteoarthritis, and the choice between simultaneous (SimBTKA) and staged (StaBTKA) procedures remains a critical decision. This meta-analysis systematically reviews and analyzes the existing literature to compare mortality outcomes associated with SimBTKA and StaBTKA.

A comprehensive search was conducted across major databases for studies reporting mortality outcomes in SimBTKA and StaBTKA. Inclusion criteria encompassed studies published up to the cutoff date of January 2023, and a total of 37 studies were included in the quantitative synthesis. Meta-analysis was performed using a random-effects model to calculate odds ratios (ORs) with 95% confidence intervals (CIs) using the Review Manage 5.4 software.

The meta-analysis included 86,333 SimBTKA cases and 115,146 StaBTKA cases. The overall mortality rate in SimBTKA was 0.66%, while StaBTKA's was 0.43%. The pooled OR for mortality in SimBTKA versus StaBTKA was 1.55 [1.16, 2.08], indicating a statistically significant higher mortality risk in SimBTKA. Our findings suggest that SimBTKA is associated with an increased risk of mortality compared to StaBTKA.

This meta-analysis provides valuable insights into the comparative mortality outcomes of SimBTKA and StaBTKA. While SimBTKA may offer potential advantages, including a single anesthesia event and shorter recovery time, clinicians should consider the increased mortality risk associated with this approach. Future research should focus on prospective studies with standardized reporting to further elucidate the nuanced factors influencing mortality outcomes in bilateral knee arthroplasty.

## Introduction and background

Osteoarthritis (OA) is one of the most prevalent types of musculoskeletal pathology and involves global degeneration of body joints, affecting articular cartilage and other surrounding tissue. It damages cartilage and remodels subarticular bone, with joint ligaments becoming lax, osteophyte formation, and decreased surrounding muscle strength. The primary symptoms are joint pain, stiffness, and joint-movement limitations, subsequently leading to a progressive reduction in the quality of life through disability [1,2]. Worldwide, approximately 8-15% of the population is affected by OA, and around 50% of people aged 75 years and older show severe osteoarthritic radiographic changes [3,4]. The 2018 Arthritis Research report estimated that 4.11 million adults (18.2%) aged 45 years and older in the United Kingdom are treated for knee OA, and 6% have a severe knee condition. In the United Kingdom, it is the most common chronic condition within primary care, and by 2030, it is predicted to be the greatest cause of disability in the general population [5,6].

According to the Kellgren-Lawrence scale of Grade 4, end-stage knee OA occurs when large osteophytes are present, joint space narrows, with severe sclerosis and definite bone contour deformity [7]. Individuals with end-stage knee OA complain that pain persists at rest and at night, which may disturb sleep, in addition to a marked and limited range of motion. Pain and limited movement are major sources of limiting physical activity and, subsequently, chronic disability [8]. Total knee arthroplasty (TKA) surgery was first reported in the 1970s and '80s [9,10]. When compared with conservative treatments for end-stage OA, pharmacological treatment is neither clinically effective for pain or pathology progression nor cost-effective. Six months of pharmacological treatment costs around €448 for one patient, which shows the financial impact on society [11]. This agrees with the study by Stan et al. [12], who assessed the cost-effectiveness of conservative and surgical treatments for late-stage knee OA. Cost-effectiveness analysis examined the ratio of direct costs to associated patient benefits. The median cost-effectiveness ratio per quality-adjusted life year was €1800 for rehabilitation versus €1268 for total knee arthroplasty [12].

Thus, TKA is a highly cost-effective intervention to manage end-stage knee OA compared with non-surgical management [12] and lies well within the range of acceptable cost-effectiveness treatments for other musculoskeletal procedures, such as lumbar spine fusion and discectomy [13]. In many patients, osteoarthritis affects the joints bilaterally, causing pain and deformity of both joints. These patients may be treated with simultaneous (SBTJA) or staged bilateral TJA (StBTJA). This study aims to systematically review current publications and estimate and compare both procedures' infection and mortality rates.

Study aim

The primary aim of this meta-analysis is to comprehensively examine and compare the mortality outcomes associated with SimBTKA versus StaBTKA. By synthesizing the existing body of literature on this topic, we aim to provide clinicians, researchers, and policymakers with a robust and evidence-based understanding of the mortality risks associated with these two surgical approaches for bilateral knee arthroplasty.

Study question

In the context of bilateral total knee arthroplasty, our study seeks to answer the following primary question: What is the comparative risk of mortality between patients undergoing simultaneous bilateral total knee arthroplasty (SimBTKA) and those undergoing staged bilateral total knee arthroplasty (StaBTKA)?

## Review

Methodology

Study Design

This is a systematic review and meta-analysis. The reporting of this meta-analysis follows the guidelines set forth by the Preferred Reporting Items for Systematic Reviews and Meta-Analyses (PRISMA) to enhance transparency and reproducibility. 

Identification of Relevant Studies

The initial phase of our meta-analysis involved a systematic and exhaustive search to identify studies comparing mortality outcomes following simultaneous (SimBTKA) and staged bilateral total knee arthroplasty (StaBTKA). A comprehensive search strategy was employed across major electronic databases, including PubMed, Embase, Scopus, and the Cochrane Library. The search encompassed studies published up to the knowledge cutoff date of January 2023.

Inclusion and Exclusion Criteria

The inclusion criteria were defined to ensure the relevance and quality of the studies. Studies were eligible if they compared mortality outcomes between SimBTKA and StaBTKA, were published in peer-reviewed journals, and were available in English. Conference abstracts, letters, and reviews were excluded. There were no restrictions based on the study design to encompass both retrospective and prospective studies.

Search Strategy

The search strategy was formulated with a combination of medical subject heading (MeSH) terms and keywords related to bilateral knee arthroplasty, mortality, and surgical approach. The search syntax was adapted to the specific requirements of each database, incorporating Boolean operators to optimize precision and recall-the search strategy aimed to cast a wide net to ensure the inclusivity of all relevant studies.

Study Selection

Two independent reviewers initially screened titles and abstracts to identify potentially relevant studies. Full-text articles were subsequently assessed for eligibility based on the predefined inclusion and exclusion criteria. Any disagreements were resolved through discussion or consultation with a third reviewer if necessary. The selection process followed the Preferred Reporting Items for Systematic Reviews and Meta-Analyses (PRISMA) guidelines.

Data Extraction

A standardized data extraction form was developed to collect relevant information from the included studies systematically. Data extraction encompassed details on study characteristics (author, publication year, country), study design, sample size, patient demographics (age, sex), surgical approach (SimBTKA, StaBTKA), and outcomes (mortality rates in both groups). Two reviewers performed the data extraction process independently to ensure accuracy and consistency.

Statistical Analysis

Data analysis and meta-analysis were conducted using Review Manager (RevMan) 5.4 software. Meta-analysis was conducted using the random-effects model to account for potential heterogeneity among the included studies. The primary outcome measure was the odds ratio (OR) with 95% confidence intervals (CIs) for mortality in SimBTKA compared to StaBTKA. 

Heterogeneity Assessment

Heterogeneity among studies was assessed using the Cochran's Q test and the I² statistic. A p-value less than 0.10 for the Q test and an I² value greater than 50% were considered indicative of substantial heterogeneity. Sensitivity analyses were conducted to explore the robustness of our findings and identify potential sources of heterogeneity. The sensitivity analysis involved recalculating the overall effect by systematically excluding one study at a time, and the pooled odds ratio (OR) and 95% confidence interval (CI) were recalculated. This approach identifies studies that substantially impact the overall effect size and assesses the consistency of our results.

Publication Bias

Publication bias was assessed using a funnel plot and Egger's test. A symmetrical funnel plot and a non-significant Egger's test would suggest minimal publication bias. Adjustments were made if significant publication bias was detected.

Results

Search Results

**Figure 1:**
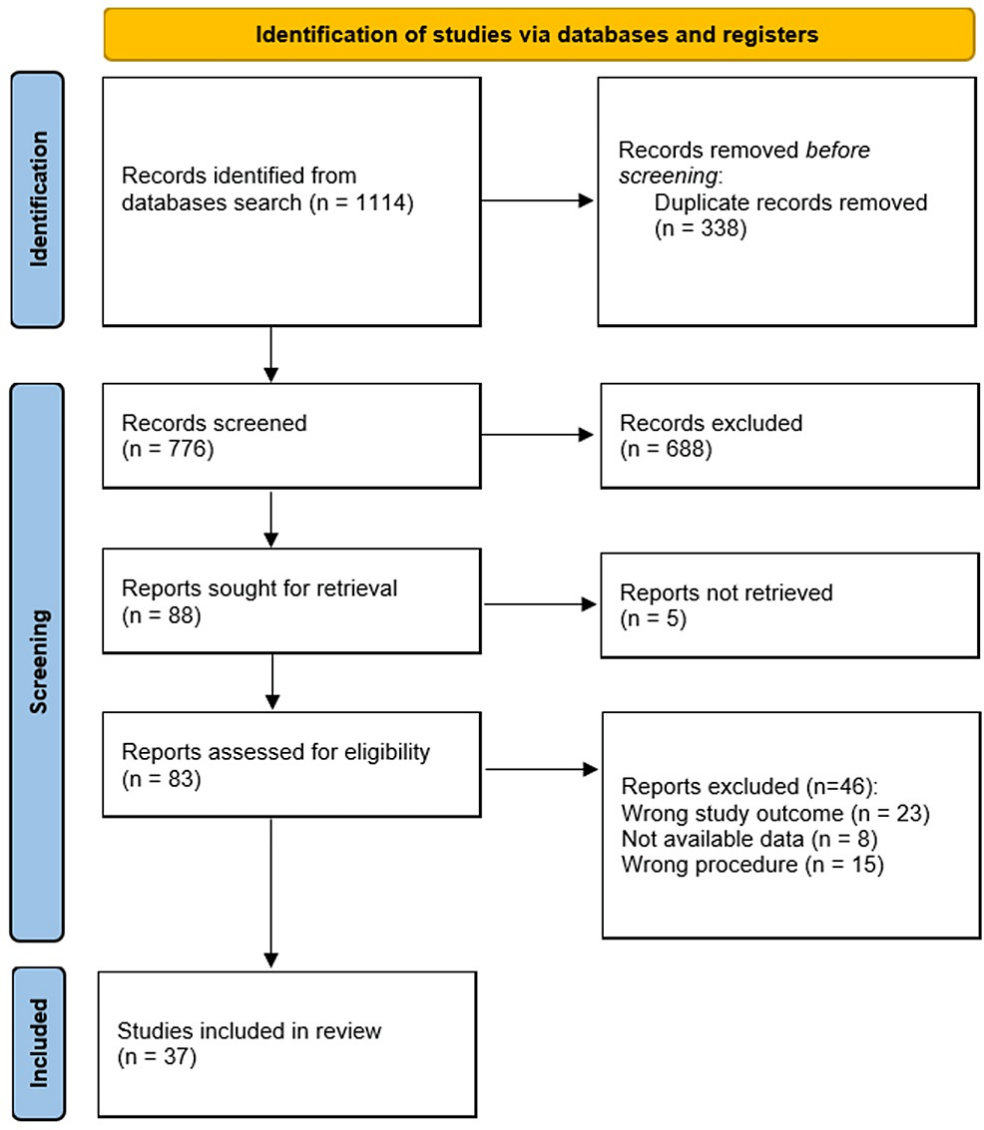
PRISMA flow diagram for a summary of the search process

The initial database search yielded 1,114 potentially relevant studies. Following the removal of duplicates, title, abstract screening, and full-text assessment, a total of 37 studies were included in the quantitative synthesis. The Preferred Reporting Items for Systematic Reviews and Meta-Analyses (PRISMA) flow diagram illustrates the study selection process (Figure 1).

The included studies spanned various countries, providing a diverse global perspective on SimBTKA and StaBTKA. The characteristics of the studies are summarized in Table 1. Noteworthy contributors to the evidence pool included the United States [14-26], Australia [27-31], and Iran [32-33], among others.

**Table 1:** Characters of the included studies and populations. NA: Not Available

Study	Country	Design	SimBTKA				StaBTKA			
			Sample size	Male, %	Age, y	Mortality, %	Sample size	Male, %	Age, y	Mortality, %
Abdelaal et al. [14]	USA	Retrospective	2725	40.30%	63.2	0.18%	1459	37.00%	67.8	0.07%
Berend et al. [15]	USA	Retrospective	35	NA	58.2	0.00%	141	NA	62.7	0.00%
Biazzo et al. [41]	Italy	Retrospective	51	25.50%	70.4	7.84%	51	37.30%	68.5	0.00%
Bini et al. [16]	USA	Retrospective	1230	41.60%	66	0.16%	2123	34.20%	67	0.00%
Bohm et al. [42]	Canada	Retrospective	6349	41%	64	0.16%	25253	39%	66	0.00%
Bolognesi et al. [17]	USA	Retrospective	8307	22%	73.3	3.32%	3788	39%	74.1	2.16%
Çelen et al. [43]	Turkey	Retrospective	168	15.47%	67.3	3.57%	63	19.04%	67.1	7.94%
Chan et al. [44]	UK	Retrospective	159	57.90%	66	0.00%	80	43.80%	66.4	0.00%
Chen et al. [34]	Singapore	Prospective	124	26.60%	62.9	0.81%	47	23.40%	61.6	0.00%
Chua et al. [27]	Australia	Retrospective	23136	53.80%	NA	0.17%	12951	50.60%	NA	0.06%
Courtney et al. [18]	USA	Retrospective	103	33%	59.4	0.00%	131	23%	64.2	0.76%
Eke et al. [45]	Turkey	Retrospective	225	15.10%	66.9	0.44%	51	24%	69.5	0.00%
Feng et al. [35]	China	Prospective	39	15.40%	64.9	2.56%	54	9.30%	64.2	0.00%
Forster et al. [28]	Australia	Retrospective	28	53.60%	66	0.00%	36	50%	68	0.00%
Ghadimi et al. [32]	Iran	Cross-sectional	60	25%	62.5	0.00%	59	16.90%	68.8	0.00%
Gill et al. [29]	Australia	Retrospective	122	37.70%	70.6	0.82%	46	50%	70.7	0.00%
Hutchinson et al. [36]	Australia	Prospective	438	56%	67	0.23%	125	38%	65	0.80%
Koh et al. [39]	South Korea	Retrospective	820	4.10%	68.6	0.24%	633	4.10%	69.7	1.89%
Lindberg-Larsen et al. [46]	Denmark	Retrospective	157	47.10%	64	0.00%	628	42.90%	66	0.96%
Mangaleshkar et al. [30]	UK	Retrospective	54	38.90%	73	7.41%	34	38.30%	71.7	0.00%
Mardani-Kivi et al. [33]	Iran	Cross-sectional	272	28.67%	NA	1.84%	391	35.03%	NA	2.05%
Meehan et al. [19]	USA	Retrospective	11445	46.10%	67.2	0.38%	23715	38.70%	67.7	0.32%
Najfeld et al. [40]	Germany	Retrospective	53	58.49%	69.6	0.00%	64	42.19%	69.7	0.00%
Niki et al. [37]	Japan	Prospective	60	16.70%	73	0.00%	60	16.70%	72.3	0.00%
Poultsides et al. [20]	USA	Retrospective	2825	37.60%	65.2	0.00%	1151	32.20%	69.5	0.00%
Richardson et al. [21]	USA	Retrospective	1637	44.20%	NA	0.00%	6110	36.68%	NA	0.00%
Ritter et al. [22]	USA	Retrospective	2050	44.20%	69.9	0.68%	152	23%	69.2	0.66%
Sarzaeem et al. [47]	Iran	Retrospective	51	9.00%	62.4	0.00%	49	10.00%	61.7	0.00%
Sheth et al. [23]	USA	Retrospective	2814	42.70%	64.9	0.28%	5177	36.60%	66.8	0.10%
Siedlecki et al. [48]	France	Retrospective	44	45.50%	69.2	2.27%	26	26.90%	70	11.54%
Sliva et al. [24]	USA	Retrospective	26	54%	59.3	0.00%	306	34.60%	67.2	0.33%
Stefánsdóttir et al. [49]	Sweden	Retrospective	1139	40.80%	70.4	0.97%	3432	37.50%	71.2	0.15%
Stubbs et al. [31]	Australia	Retrospective	61	NA	68.5	0.00%	38	NA	71.3	0.00%
Tsay et al. [38]	USA	Retrospective	27301	43.20%	65.8	0.94%	45419	37.40%	66.6	0.61%
Walmsley et al. [25]	UK	Retrospective	826	NA	NA	0.97%	1796	NA	NA	0.28%
Wyles et al. [26]	USA	Retrospective	188	42%	61	0.00%	242	36%	72	0.00%
Yoon et al. [50]	South Korea	Retrospective	119	5.90%	70	0.00%	119	5.90%	70	0.00%
Overall			Total SimBTKA: 86333	Average SimBTKA Mortality incidence		0.66%	Total StaBTKA: 115146	Average StaBTKA Mortality incidence		0.43%

The studies employed diverse research designs, ranging from retrospective analyses to prospective and cross-sectional investigations. This methodological diversity enhances the robustness of the meta-analysis by incorporating findings from various study types. Notable retrospective studies include those conducted by Abdelaal [14], Berend [15], and Bini [16], while prospective designs were employed by Chen [34], Feng [35], Hutchinson [36], and Niki [37]. Additionally, cross-sectional studies were conducted by Ghadimi [32] and Mardani-Kivi [33].

The aggregated data revealed substantial variation in population sizes across studies. For SimBTKA, sample sizes ranged from smaller cohorts, such as the study by Forster [28] with 28 cases, to extensive datasets like the one compiled by Tsay [38] with 27,301 cases. Similar variations were observed in StaBTKA studies, from the smaller cohort of Berend [15], with 141 cases, to the extensive dataset of Tsay [38], with 45,419 cases. Sex distribution also varied, with male percentages ranging from as low as 4.10% in the study by Koh [39] to as high as 58.49% in the study by Najfeld [40].

The age of the populations included in the studies demonstrated a considerable range, reflecting the diversity of patients undergoing bilateral knee arthroplasty. For instance, the age of patients undergoing SimBTKA ranged from a mean of 59.3 years in Sliva [24] to 73.3 years in Bolognesi [17]. Similarly, for StaBTKA, age ranged from a mean of 61 years in Wyles [26] to 74.1 years in Bolognesi [17].

The meta-analysis synthesized mortality data across the included studies, revealing important insights as it included 86,333 SimBTKA cases and 115,146 StaBTKA cases. The overall mortality rate in SimBTKA was 0.66%, while StaBTKA's was 0.43%.

For SimBTKA, mortality rates varied, with the lowest observed in studies like Berend [15], Bini [16], and Walmsley [25] at 0.00%, while the highest was reported in Biazzo [41] at 7.84%. For StaBTKA, mortality rates ranged from 0.00% in studies by Berend [15], Chen [34], and Tsay [38] to 2.05% in Mardani-Kivi [33].

Quantitative Data Synthesis

**Figure 2:**
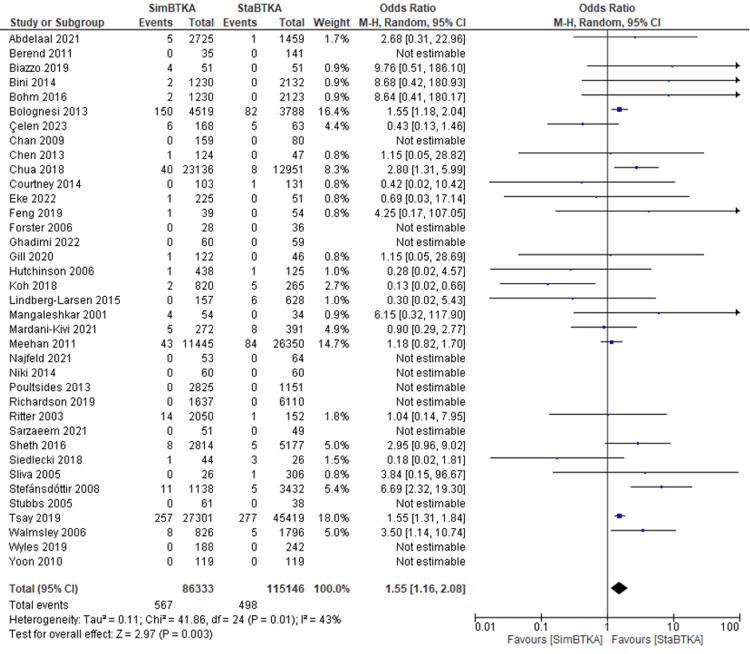
Forest plot of mortality among SimBTKA versus StaBTKA groups. Pooled OR calculated by Random Effects meta-analysis of the included studies [14-50].

The forest plot in Figure 2 visually represents the meta-analysis comparing mortality rates between SimBTKA and StaBTKA groups across the included studies. 

The summary estimate for the overall effect is an odds ratio of 1.55 [1.16, 2.08], indicating a statistically significant difference in mortality between SimBTKA and StaBTKA groups. The heterogeneity test revealed moderate heterogeneity (I² = 43%), suggesting variability in the study outcomes. 

The sensitivity analysis did not reveal significant alterations in the overall effect estimate upon excluding individual studies. Excluding any single study did not significantly alter the pooled OR or its 95% CI. The lowest and highest point estimates for the OR after excluding any single study were 1.44 and 1.64, respectively, within the original 95% CI (1.16, 2.08). This suggests that no single study drives the observed heterogeneity and that the overall effect size is relatively robust to individual study variations. It is noteworthy that when we excluded two studies with potentially influential data points [39,48], the pooled analysis heterogeneity became non-significant (Tau² = 0.05, Chi² = 29.71, P = 0.13, I² = 26%), while this did not alter the direction or significance of the pooled estimate. This suggests that the overall effect size may be more robust than initially indicated, particularly if outliers are excluded. However, it is important to interpret the results for clinical practice cautiously.

Examining the forest plot, several studies, such as Abdelaal [14], Biazzo [41], Bini [16], and Bohm [42], showed SimBTKA mortality rates ranging from 0.90% to 1.70%, while StaBTKA mortality rates were predominantly 0.00%, indicating a favorable outcome for staged procedures. Notably, Bolognesi [17] reported a higher mortality rate for SimBTKA (16.40%) compared to StaBTKA (2.16%).

Studies like Chua [27] and Tsay [38] showed higher odds of mortality in SimBTKA compared to StaBTKA, with ORs of 2.80 [1.31, 5.99] and 1.55 [1.31, 1.84], respectively. On the other hand, studies like Koh [39] and Siedlecki [47]demonstrated lower odds for SimBTKA mortality, with ORs of 0.13 [0.02, 0.66] and 0.18 [0.02, 1.81], respectively.

Publication Bias

**Figure 3:**
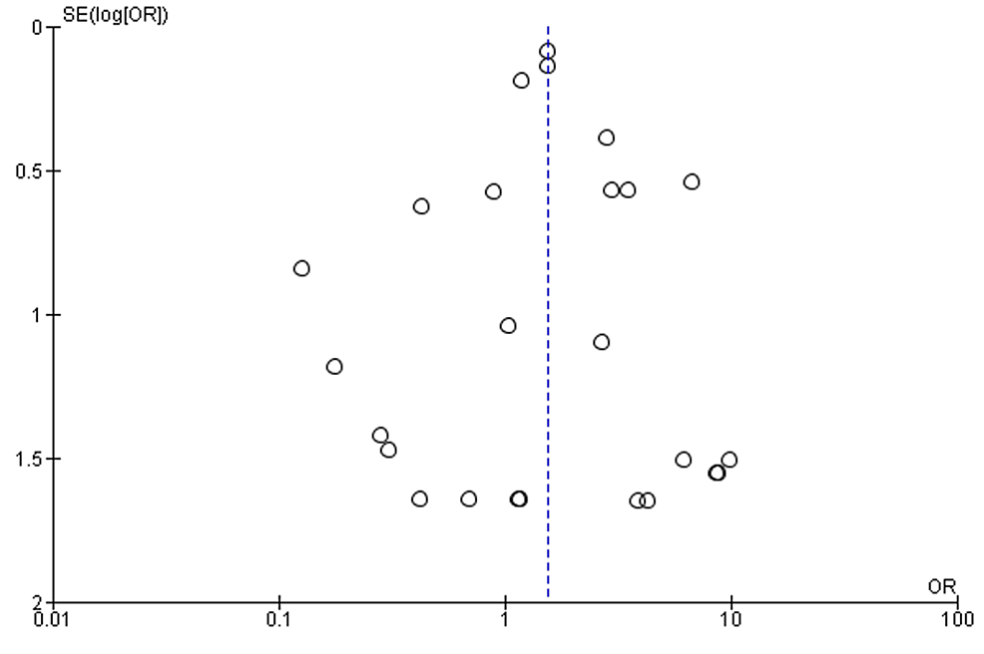
Funnel plot of mortality among SimBTKA versus StaBTKA groups.

Figure 3 presents a funnel plot, a graphical tool used to assess potential publication bias in the included studies comparing mortality rates between SimBTKA and StaBTKA groups. The symmetrical distribution of points in the funnel plot is reassuring and suggests that there might be minimal publication bias within the meta-analysis.

Discussion

Bilateral total knee arthroplasty (BTKA) is a significant intervention for individuals suffering from bilateral knee osteoarthritis, aiming to alleviate pain and improve function simultaneously. The decision between simultaneous (SimBTKA) and staged (StaBTKA) procedures remains a critical consideration, impacting clinical outcomes and healthcare resource utilization. Our meta-analysis sought to shed light on this dilemma by synthesizing data from 37 studies, providing a comprehensive overview of mortality outcomes associated with SimBTKA and StaBTKA.

Our findings indicate a statistically significant difference in mortality between SimBTKA and StaBTKA, with an odds ratio (OR) of 1.55 [1.16, 2.08]. The overall mortality rate for SimBTKA was 0.66%, while StaBTKA's was 0.43%. This implies a higher mortality risk associated with SimBTKA. Interpreting these results is crucial, considering the potential factors contributing to this difference.

The variability in mortality rates across studies underscores the complexity of this decision-making process. Individual studies, such as those by Chua et al. [27] and Tsay et al. [38], reported higher mortality rates in SimBTKA, aligning with our meta-analysis results. However, some studies, like Lindberg-Larsen et al. [46], report no mortality in SimBTKA, suggesting the existence of specific patient populations or surgical contexts where SimBTKA may be a safe option.

The observed heterogeneity in our analysis (P = 0.01, I² = 43%) emphasizes the diversity in patient populations, surgical techniques, and healthcare systems among the included studies. Sensitivity analysis showed that excluding individual studies did not affect the overall heterogeneity or the overall effect significance. This diversity highlights the need for cautious interpretation and underscores the importance of considering study design, geographical variations, and sample size in evaluating mortality outcomes.

Geographic variations were evident in our analysis, with studies from different regions contributing to the observed heterogeneity. For example, studies from Iran reported no mortality in either group, while studies from the United States reported varying mortality rates. Variations may influence these differences in patient characteristics, healthcare infrastructure, and perioperative practices across regions.

Considering the impact of sample size, larger studies tended to report lower mortality rates in SimBTKA. The study by Tsay et al. [38], with a massive sample size of 27,301 SimBTKA cases, reported a mortality rate of 0.94%. In contrast, smaller studies like Berend et al. [15], with 35 cases, reported no mortality. The influence of sample size on outcomes emphasizes the need for cautious interpretation of results from smaller studies.

The increased mortality risk associated with SimBTKA could be attributed to several factors. Simultaneous procedures impose greater physiological stress on the patient, potentially leading to increased perioperative complications. The complexity of managing simultaneous bilateral interventions may contribute to a higher risk of adverse events. The findings align with those of Bolognesi et al. [17], who reported a mortality rate of 3.32% in SimBTKA compared to 2.16% in StaBTKA, emphasizing the importance of careful patient selection and perioperative management in SimBTKA.

Clinical Implications and Future Directions

The findings from this meta-analysis have important implications for surgeons, patients, and healthcare providers involved in the decision-making process for bilateral knee arthroplasty. SimBTKA, while potentially offering advantages such as a single anesthesia event and shorter overall recovery time, should be approached cautiously, particularly in patients with significant comorbidities or increased surgical risk.

Future research should focus on well-designed prospective studies with standardized reporting of patient characteristics, perioperative management, and long-term outcomes. Exploring specific patient subgroups, such as age, comorbidity profiles, and socioeconomic factors, may provide more nuanced insights into the optimal approach for different populations.

## Conclusions

In conclusion, our meta-analysis reveals a higher mortality risk associated with SimBTKA than StaBTKA. While staged procedures appear to carry a lower mortality risk, individual patient characteristics, surgeon expertise, and healthcare infrastructure should guide the decision-making process. The complexity of this decision necessitates a personalized approach, taking into account the unique attributes of each patient to optimize outcomes in bilateral knee arthroplasty.
